# Efficacy of Vehicle Scanning Methods in Estimating the Mode Shapes of Bridges Seated on Elastic Supports

**DOI:** 10.3390/s23146335

**Published:** 2023-07-12

**Authors:** Kultigin Demirlioglu, Semih Gonen, Emrah Erduran

**Affiliations:** 1 Department of Built Environment, Oslo Metropolitan University, P.O. Box 4, NO-0130 Oslo, Norway; kultigin@oslomet.no; 2 Department of Civil and Environmental Engineering, Universitat Politècnica de Catalunya (UPC-BarcelonaTech), 08034 Barcelona, Spain; semih.gonen@upc.edu

**Keywords:** structural health monitoring, vehicle scanning methods, bridges, mode shape estimation, elastic support, road roughness

## Abstract

This study systematically assesses the efficacy of the vehicle scanning methods (VSM) in accurately estimating the mode shapes of bridges seated on elastic supports. Three state-of-the-art VSM methods are employed to obtain the mode shapes of bridges using the vehicle data during its travel. Two of the evaluated methods use a signal decomposition technique to extract the modal components of the bridge from the contact point of the response while the third one relies on the segmentation of the measured signals along the bridge deck and applying an operational modal analysis tool to each segmented signal to estimate the mode shapes. Numerical analyses are conducted on one single- and one two-span bridge, considering smooth and rough road profiles, different vehicle speeds, and presence of lead vehicle. The accuracy of the numerical models used in developing and assessing vehicle scanning models is tested, and the results of the study demonstrate the method using a half-car vehicle model and signal decomposition technique shows robustness against increasing vehicle speeds and road roughness while the method applying the segmentation of the measured signals provides relatively accurate mode shape estimates at the bridge edges at low speed, although the three methods have their limitations. It is also observed that simplified bridge and vehicle models can hide potential challenges that arise from the complexity of actual vehicle and bridge systems. Considering that a significant number of bridges worldwide are built on elastic supports, the practical success of vehicle scanning methods depends on their ability to handle elastic boundary conditions with reliability. Therefore, the article provides valuable insights into the capabilities and limitations of the current vehicle scanning methods, paving the way for further advancements and refinements in these techniques.

## 1. Introduction

Constant exposure of bridges to various actions such as environmental conditions, natural hazards, and excessive loads can result in structural degradation and failure, as evidenced by recent incidents of bridge collapses. As a result, implementation of structural health monitoring (SHM) systems for the evaluation and maintenance of transport infrastructure has been gaining attention. In the majority of SHM applications, bridges are monitored by mounting the sensors directly on the structure. Hence, these methods are called direct measurement methods. Each direct measurement application requires separate data acquisition systems and, often, numerous sensors [1,2,3,4,5,6,7,8,9,10,11,12]. Thus, this conventional approach incurs exorbitant costs when the entire transportation network is to be monitored continuously. In the last couple of decades, a new approach called indirect method or vehicle scanning method (VSM) has emerged, which involves using a passing vehicle equipped with vibration sensors to perform bridge monitoring. Yang et al. [13] proposed this indirect method first, and this study aimed to extract the modal parameters of the bridge from the acceleration response recorded on the moving vehicle. VSM promises efficient and low-cost inspection for the condition assessment of bridges without the requirement of instrumentation of each bridge. Thus, VSM has been further developed to extract a wide range parameters such as natural frequencies [13,14,15], mode shapes [16,17], damping ratios [18,19], and damage occurring on the bridge [20,21,22,23]. Among these parameters, mode shapes are particularly important because it serves as a key indicator for creating and updating finite element (FE) models of bridges as well as detecting local and global damage.

Several researchers attempted to develop methods to estimate the bridge mode shapes from the vibrations recorded on vehicle-mounted sensors. The early studies focused on the resolution of the vibrations in the time–frequency domain. As an example of these studies, Zhang et al. [16] attempted to construct the mode shapes of bridges using the amplitude history of the short-time Fourier transform (STFT) applied to the acceleration response of the vehicle passing over a simply supported bridge with a smooth profile. In order to increase the vibration amplitudes, the vehicle was excited using tapping forces with frequencies close to the natural frequency of the bridge. Later, Kong et al. [17] proposed a method utilizing STFT and a special test vehicle consisting of a tractor and two trailing trailers to extract the bridge modal characteristics more accurately by eliminating the driving-related frequencies and road roughness effects. Unlike Zhang et al. [16], they included the effect of road roughness in numerical simulations. To eliminate the effect of road roughness, the residual responses were used by subtracting one trailer response from the other, both measured at the same location on the bridge during their passage. Later, Kong et al. [24] verified the methodology proposed in [17] using field test data. This study demonstrated that the extraction of bridge mode shapes was not satisfactory because of the limitation of the STFT method. More recently, Jian et al. [25] employed wavelet transform in combination with an iterative multiplication to identify the frequencies and mode shapes of bridge structures. Zhang et al. [26] mentioned that neither STFT nor continuous wavelet transform could achieve a good frequency resolution. They developed a modified time–frequency analysis method to evaluate the non-stationary instantaneous frequencies and reconstruct the mode shapes. Yang et al. [27] proposed a method to estimate the damping ratios and mode shapes, and to evaluate the element stiffness using STFT, assuming that the fundamental mode shape amplitude peak is located at the midspan. Furthermore, the method was validated through field testing on the Li-Zi-Wan bridge in Chongqing.

Another approach used by several researchers relies on the segmentation of the measured signals along the bridge deck and applying an operational modal analysis (OMA) tool to each segmented signal to estimate the mode shapes. One of the first examples of such applications can be found in Oshima et al. [28], which used singular value decomposition (SVD) to extract mode shapes from a passing vehicle. Malekjafarian and O’Brien [29] applied short-time–frequency domain decomposition (STFDD) to dynamic responses of quarter-cars traveling over a bridge to estimate the bridge mode shapes. In a subsequent study [30], they enhanced the resolution of identified mode shapes by combining STFDD with multiple laser measurements from passing vehicles. Li et al. [31] used acceleration responses measured on both a stationary (reference) and a moving car, employing stochastic subspace identification (SSI) for mode shape identification. Applications that are based on the same philosophy can be found in [32,33,34], which aim to estimate the mode shapes of the bridge utilizing a crowdsourcing-based methodology through a non-parametric sparse matrix completion.

The final approach used in mode shape estimation is based on using a signal decomposition technique to extract the bridge’s modal components from the vehicle’s acceleration response and determine the mode shapes by applying the Hilbert transform (HT) to the obtained components. Yang et al. [35] conducted the pioneering work using this approach where the modal components of bridge are extracted using a digital filter such as band-pass filter [35,36,37,38], elliptic filter [39], or a recently introduced two-peak spectrum idealized filter [40]. Alternatively, a blind source separation algorithm such as empirical mode decomposition (EMD) [41] or variational mode decomposition (VMD) [42] can be used to extract the narrow-band components of the vibrations measured on the vehicle that correspond to the bridge modal frequencies. Some researchers [36,41] combined this mode shape identification technique with impact excitation, as described in [16]. Tan et al. [37] proposed an algorithm for adjusting the shifted mode shapes caused by bridge damping and applied it to detect bridge damage using mode shape squares [38]. Furthermore, Yang et al. [43] argued that the contact-point (CP) response does not contain vehicle frequencies that hamper identifying the bridge mode shapes and frequencies. This observation was verified through numerical simulations [43] and an in situ test [44] and use of CP response to estimate the mode shapes has become more popular afterwards [23,39,42,43,44,45,46].

These studies made significant contributions to the mode shape identification of bridges using indirect measurements. However, most of the existing studies in this field have only utilized simplified car models simulated as a single sprung mass, which does not account for the pitching action of real vehicles. Only a few studies have employed two-axle vehicle models that consider the pitching action to scan the bridge mode shapes [24,30,32,40,41,42]. More importantly, most of the numerical studies on mode shape identification have tested the accuracy of their methods on simply supported bridge models. In contrast, the majority of real-world bridges are supported by elastic springs (bearings) [47] at the support points, which introduces additional challenges for mode shape identification. Consequently, a fundamental question arises: to what extent can the state-of-the-art vehicle scanning methods successfully construct the mode shapes of bridges that are seated on elastic supports?

To address this gap in the literature, this paper, for the first time, investigates the effectiveness of three state-of-the-art vehicle scanning methods [31,39,42] in estimating the mode shapes of bridges seated on elastic supports. For this, we developed numerical models of two bridges, one single- and one two-span, that are seated on elastic supports. Then, vehicle-bridge interaction analysis was carried out in ABAQUS computational environment to compute the contact point and vehicle accelerations. The numerical analyses were conducted for three different scenarios for each bridge: (i) assuming a smooth road profile, (ii) considering the effects of road roughness, and (iii) sending a lead vehicle to increase the vibration amplitudes and to attenuate the adverse effects of road roughness. We then used the methods summarized in [31,39,42] separately to estimate the bridge mode shapes for each scenario. We paid special attention to the modal displacement estimates at the edges of the bridge, i.e., over or close to the elastic supports, because none of the three methods was previously tested on bridges that are seated on elastic supports, which have very different modal amplitudes at the support locations compared to their simply supported counterparts. As a secondary objective, the speed of the vehicle was varied to investigate the effect of this parameter on the efficacy of the VSMs to estimate the bridge mode shapes.

The novelty of this study lies in its pioneering approach, as it is, to the best of our knowledge, the first article that systematically assesses the efficacy of vehicle scanning methods in accurately estimating the mode shapes of bridges seated on elastic supports. By shedding light on the strengths and weaknesses of state-of-the-art VSMs, this research contributes to the broader understanding of their performance and sets the stage for the development of enhanced methods in the future.

## 2. Vehicle Scanning Methods

Although some researchers used time–frequency domain techniques [16,17,24,25,26,27], the majority of the most recent vehicle scanning methods in the literature apply two mode shape identification approaches to construct the bridge mode shapes: (i) using signal segmentation with an OMA tool [28,29,30,31,32,33,34], and (ii) using signal decomposition together with Hilbert transform [35,36,37,38,39,40,41,42]. This study involves three of the most recent VSM methods proposed in the literature for estimating mode shapes. The first method, proposed by Li et al. [31], performs the segmentation of the measured signals along the bridge deck and uses stochastic subspace identification (SSI) as an OMA tool. The other two methods construct the mode shapes through signal decomposition processed by Hilbert transform, but differ in terms of the vehicle model and the signal decomposition tool used. Yang et al. [42] uses a half-car model with two accelerometers positioned at the back and front of the car and employs variational mode decomposition (VMD) as the signal decomposition tool. On the other hand, Yang and Wang [39] employs a quarter-car model simulated using a single sprung mass with a single accelerometer and uses an elliptic filter as the signal decomposition tool. The half-car model with two accelerometers used in [42] aims to mitigate the adverse effects of road roughness while also considering the pitching effect of the vehicle.

To facilitate easier reading, from this point on, the method developed by Li et al. [31] will be referred to as the “reference-based SSI method”, while the methods by Yang et al. [42] and Yang and Wang [39] will be referred to as “half-car method” and “elliptic filter method”, respectively.

**Figure 1:**
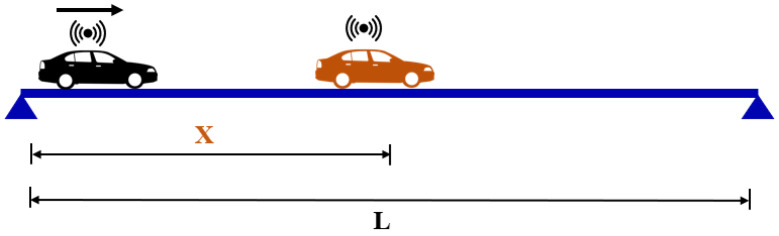
Schematic description of vehicle scanning methods.

To explain the principles of the three methods, we refer to the schematic illustration of a bridge and two instrumented-cars shown in Figure 1. This figure illustrates the common properties of the numerical models that were used in the development of the three methods: a simply supported beam of length *L* and an instrumented vehicle that travels on the bridge. In addition, there is also a second instrumented vehicle in Figure 1 depicted with the orange color. This vehicle is used only in the reference-based SSI method and is stationary. In the following paragraphs, we briefly summarize the three methods. For further details, the readers are referred to the original articles [31,39,42]. In order to gain insights into the theoretical background of vehicle scanning methods, the readers are referred to the articles that provide detailed information on the governing equations of motion for quarter-car [13,31] and half-car [40,42] vehicle models.

### 2.1. Reference-Based SSI Method

In the reference-based SSI method [31], two instrumented vehicles are used to measure the dynamic response of the bridge. One of the vehicles is located at a pre-selected location on the bridge deck, acting as a reference sensor (orange car in Figure 1) while the other instrumented vehicle travels on the bridge with a constant velocity measuring the dynamic response. This method requires dividing the bridge into *N* physical segments. The signals measured at each segment of the bridge are assumed to be similar to signals measured at separate physical sensors used in direct monitoring. As such, the reference-based SSI method [31] emulates a direct monitoring campaign where a reference sensor remains stationary while another sensor is moved to different segments of the bridge.

Once the measurements are completed, the stochastic subspace identification algorithm (SSI) is applied to the data recorded at each segment to obtain the local mode shape values at each segment. In order to estimate the global mode shape from these local mode shape values, a progressive rescaling procedure is applied. This is achieved by rescaling the local mode shape values obtained from the moving vehicle at each segment with respect to that at the reference location where the stationary vehicle is located. If the reference sensor is located at the $i^{th}$ segment of the bridge, the local mode displacement values for this segment are computed from the signals measured on the moving and reference sensors concurrently. Therefore, these modal displacement values can be treated as the global mode displacement values for that segment; $\Phi_{i}=\Phi_{ref}$. The global mode shape values of all the segments can then be obtained using the rescaling factor:

(1) $$\Phi_{j}=\frac{\Phi_{ref}}{\phi_{j,ref}}\phi_{j,j}\;\;\;\;\;\;\;\left(j=1,2,...N\right)$$

In Equation (1), $\Phi_{j}$ is the global modal displacement value for the $j^{th}$ segment, $\phi_{j,j}$ is the local modal displacement value from the moving sensor, and $\phi_{j,ref}$ is the local modal displacement from the reference sensor obtained as the moving vehicle travels on the $j^{th}$ segment of the bridge. Once the global modal displacement values for each segment are computed, they are then combined to construct the mode shape.

### 2.2. Elliptic Filter Method

Recently, Yang and Wang [39] proposed an improved vehicle scanning method for the identification of modal frequencies and mode shapes of bridges. This method, unlike the other two methods in this study, considers damping in bridges. As such, in [39], the method was tested on bridges with 1.5% and 3% viscous damping. Effect of road roughness was investigated as well. The elliptic filter method follows a similar approach to the methods proposed in [35,42] but uses an elliptic filter to decompose the response of the vehicle to its modal components instead of the VMD method that was adopted in [42]. The method was developed and validated using numerical models [39], where the vehicle was modeled as a single sprung mass simulating a quarter car. The main steps of the elliptic filter method can be summarized as:

1. Record the dynamic response of the vehicle as it is crossing the bridge of interest.
2. Compute contact point (CP) response from the accelerations recorded on the vehicle.
3. Compute the Fourier amplitude spectrum (FAS) of the CP response to determine the natural frequencies of the bridge.
4. Decompose the CP response into its modal components using an elliptic filter, which is designed based on the natural frequencies of the bridge and the signal strength to obtain the narrow band signal.
5. Calculate the analytic signal from the narrow band signal using the Hilbert transform (HT) and construct the mode shape of the bridge from the instantaneous amplitude of the HT.

In the referenced study [39], the proposed method was shown to be very accurate in estimating the modal properties of the bridge when no damping and road roughness was considered. However, when these parameters were introduced, the accuracy of the method was observed to decrease [39]. The parameters of the elliptic filter were found to significantly impact the method’s accuracy. Yang and Wang [39] optimized the values of these parameters for each of the four cases they have considered but did not provide a structured approach or criteria for this purpose. Finally, the method does not address removing the adverse effects of road roughness and relies solely on the elliptic filter for this.

One of the most significant parameters of the elliptic filter method is the design of the elliptic filter that will be used to decompose the CP response into its modal components. This filter needs to ensure that the peak and valley of each mode are accurately captured. In this study, we used the elliptic filter parameter values, including the bandwidth, passband, and stopband ripples that are recommended in [39].

### 2.3. Half-Car Method

The method developed by Yang et al. [42] differs from most of the vehicle scanning methods because it uses a half-car model [48] instead of a single-degree-of-freedom system to simulate the behavior of the vehicle. This allows considering the rotational (pitching) action of the vehicle, which can potentially bring in additional disturbance to the vehicle’s spectral response. Apart from the use of a multi-degree-of-freedom system to model the car, the method builds on the previous studies [35,49] that use Hilbert transform and variational mode decomposition (VMD) methods to extract the bridge frequencies and mode shapes. As mentioned earlier, the half-car method is very similar to the elliptic filter method that was summarized in Section 2.2. The only differences between the two methods are in steps two and four of the elliptic method. In step two, the contact point of both the front and back axles of the vehicle is computed using the backward procedure, which is summarized in [42]. In step four, the contact point responses are decomposed using variational mode decomposition, instead of an elliptic filter, to obtain the narrow band signal, which is then used to construct the estimated mode shapes using HT. Yang et al. [42] also recommend the use of band-pass filter prior to the decomposition of contact point responses by VMD in order to remove the undesired frequencies, such as the driving frequency.

Finally, in this method, the effect of road roughness is eliminated by using the residual response between the front and back contact points, which is obtained by subtracting the rear contact response from the front contact response. Through numerical analysis, Yang et al. [42] demonstrated that the half-car method can effectively capture the mode shapes of single-, two-, and three-span bridges that are hinged at the support points even when road roughness is present. However, the damping in the bridges was ignored in the numerical models while the damping of the vehicle is considered.

## 3. Numerical Models

**Figure 2:**
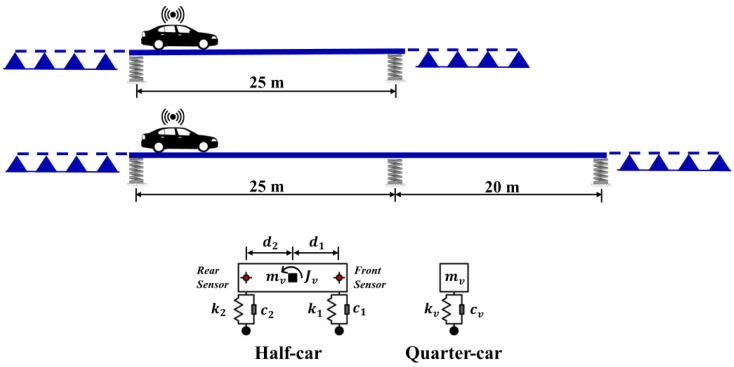
Bridge and vehicle models.

We evaluated the capabilities of VSMs in estimating mode shapes on two bridges. Figure 2 presents an overview of the two bridges along with the numerical models of the car models used in the study. The first bridge is a 25 m long single-span bridge while the other is a two-span bridge with span lengths of 25 m and 15 m. An unsymmetrical bridge was chosen on purpose because it potentially represents a more challenging task for the VSM methods and is common in practice. The bridges were modeled in ABAQUS software using Bernoulli beam elements discretized at intervals of 0.5 m. Table 1 presents the properties of the bridges and vehicles. We aim for the numerical models of the bridges used to test the efficacy of the VSMs to be as realistic as possible. Therefore, supports were modeled using elastic springs, which emulate the behavior of bearings that are often used in bridges to attenuate the thermal and seismic effects. The supports were assumed to be fixed in the longitudinal and transverse directions as the stiffness in these directions does not have a significant impact on the vertical behavior of the bridge. Although the supports in the existing bridge were shown to have some rotational stiffness due to several reasons such as aging [50], these effects were ignored, and the supports were modeled as free to rotate. Since the main novelty of the article lies in the effect of the elastic supports at the ends of the bridge, the value of its stiffness plays a crucial role. Most of the commercially available elastomeric bearings, which is the most common bearing type used in bridges, have a vertical stiffness between $1\times 10^{5}$ kN/m and $1\times 10^{7}$ kN/m [47]. We conducted a sensitivity study to select a value in this range and, as expected, the lower bound of the range, i.e., $k_{v}=1\times 10^{5}$ kN/m, provided the highest modal displacements. Since this case, arguably, provides the most critical situation in terms of estimating the mode shapes at the edges of the bridge, $k_{v}=1\times 10^{5}$ kN/m was selected to be used in the analysis. The boundary conditions at each support of the bridges were kept constant with an elastic spring coefficient of $1\times 10^{5}$ kN/m in all the numerical simulations.

Two different vehicle models were used to be able to apply all three methods to extract the bridge mode shapes. For the reference-based SSI and elliptic filter methods, the vehicles were modeled as a single sprung mass while a two-axle model was used for the half-car method; Figure 2. For the reference-based SSI method, a reference vehicle that is identical to the moving vehicle is modeled as a stationary element on the bridge as depicted in Figure 1.

Finally, rigid beams were modeled at both ends of the bridge to emulate the bridge approaches. When vehicle behavior is simulated using a quarter-car model, these approaches have no function as the car travels from one support to the other. However, when the half-car model is used, the car starts its trip when the front axle is positioned at the first support and continues until the rear axle reaches the final support. In this scenario, the rear axle travels on the approach at the beginning of the car’s motion, while the front axle does the same at the end.

**Figure 3:**
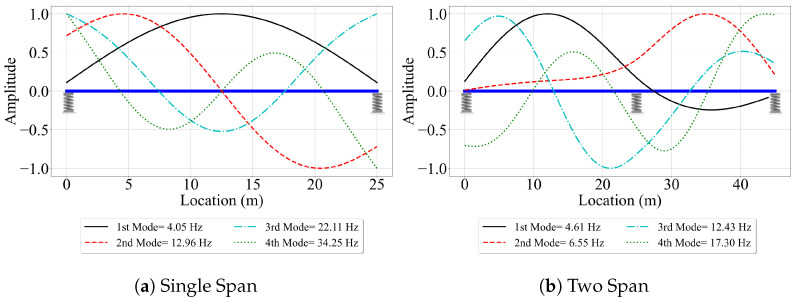
The FE mode shapes and frequencies of the (**a**) single-span bridge and (**b**) two-span bridge.

The mode shapes and modal frequencies of the first four mode shapes of the bridges computed from eigen-value analysis are presented in Figure 3. These shapes and frequencies are designated as “exact” values and are used to evaluate the efficacy of the VSMs. The effect of elastic support on the bridge mode shapes is evident from Figure 3. Especially, the higher modes have relatively high modal displacements at the support locations, which would not have been the case should the bridge have been modeled using pin supports.

In the original articles, Li et al. [31] and Yang et al. [42] ignored the damping in the bridges while developing the reference car and half-car methods, respectively. On the other hand, Yang and Wang [39] considered the bridge damping and demonstrated, to a certain extent, the potential impacts of the bridge damping on vehicle scanning methods. In this article, bridge damping was not considered as its effect was previously shown to be minimal in the forced-vibration phase of the vehicle–bridge interaction [31,35]. Vehicle damping was considered via viscous dampers, as shown in Figure 2.

Road roughness is a major source of dynamic excitation in vehicles. As a vehicle travels over a rough road surface, it is subjected to forces caused by the uneven road surface that affects its behavior and dynamic response polluting the accelerations recorded on the vehicle. To account for the road roughness, we numerically generated a road roughness profile following the procedure described below.

Dodds and Robson [51] proposed the power spectral density (PSD) functions to generate the road surface roughness that is assumed to be a zero-mean stationary Gaussian random process [51]. This function provides the amplitudes of a surface’s roughness as a function of the spatial frequency of the roughness. Spatial frequency is the inverse of the wavelength of the roughness features. According to ISO-8608 [52], the one-sided PSD function, G(n), is described in Equation (1).

(2) $$G\left(n\right)=G\left(n_{0}\right){\left(\frac{n}{n_{0}}\right)}^{-2}\;\;\;\;\;\;\;\;n_{\min}<n<n_{\max}$$

where $G(n_{0})$ is the roughness coefficient that represents the degree of roughness in the road classification ranging from Class A to Class H. In this study, $G(n_{0})$ is adopted as 16 × 10${}^{-6}$ m${}^{3}$, which accounts for Class A profile in the road classification [52], $n_{0}=0.1$ m${}^{-1}$ is the reference spatial frequency. $n_{\min}=0.01$ m${}^{-1}$ and $n_{\max}=10$ m${}^{-1}$ are the lower and upper spatial frequency limits, respectively. *n* is the spatial frequency value increased incrementally by Δn=0.01 m${}^{-1}$ in the range between $n_{\min}$ to $n_{\max}$. Road roughness irregularities are generated using the sum of a series of harmonics as described in Equation (3).

(3) $$r\left(x\right)=\sum_{i=1}^{N}\sqrt{2G(n_{i})\Delta n}\;cos\left(2\pi n_{i}x+\varphi_{i}\right)$$

where $\varphi_{i}$ is the random phase angle that varies between 0 and 2π, *x* denotes a location on the surface where the irregularity r(x) is defined, and *N* represents the number of spatial frequencies, which is calculated as follows:

(4) $$N=\frac{n_{\max}-n_{\min}}{\Delta n}+1$$

**Figure 4:**
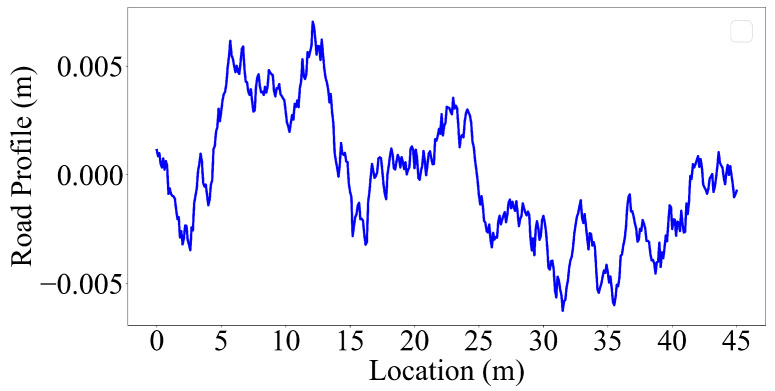
The road roughness profile generated for Class A.

The road roughness profile was generated at 10 mm intervals along the 45-m two-span bridge, as shown in Figure 4. Under the conditions where road roughness was taken into account, the initial 25-m section of the road profile was incorporated into the single-span bridge, while the full 45-m profile was utilized for the two-span bridge.

## 4. Numerical Analysis

To investigate the efficacy of the VSMs in estimating the mode shapes of the bridges, we virtually drove the vehicle on both bridges with speeds of 2.5 m/s, 5 m/s, and 10 m/s. The Newmark method with parameters α=0.5 and β=0.25 and a time step of dt=0.001 s were used in the numerical solution. The contact between the vehicle and the bridge was not lost during the analyses.

For each bridge, we considered three separate cases to investigate the effect of different parameters on the efficacy of VSMs. In the first case, we assumed a smooth road profile for the bridge. In the second case, road roughness was incorporated into the numerical model. In the last case, we introduced the effect of existing traffic by sending a lead truck to pre-excite the bridge before the instrumented vehicle crossing. The lead vehicle was assumed to be a 5t truck and was modeled as a single-degree-of-freedom system. In the following paragraphs, we first focus on the single-span bridge considering all three cases. Afterwards, we shift our focus to the two-span bridge.

### 4.1. Single-Span Bridge

Before starting to evaluate the efficacy of the three methods in estimating the mode shapes of the single-span bridge, we conducted a sensitivity study on the smooth bridge to be able to determine the optimum number of segments that will be used in the reference-based SSI method because this method was previously shown to be sensitive to the number of segments used in the analysis [31]. This sensitivity stems from the fact that having too few segments can lead to insufficient spatial resolution in mode shape estimation while having too many segments can cause insufficient data points in each segment rendering mode shape identification using stochastic subspace identification method difficult. Thus, we conducted a sensitivity study to optimize the number of segments used for the reference-based SSI method to ensure that we have the best possible results from this method when we assess its efficacy. For this, we divided the single-span bridge into five, eight, and ten segments and applied the method proposed in [31] and summarized in Section 2.1.

**Figure 5:**
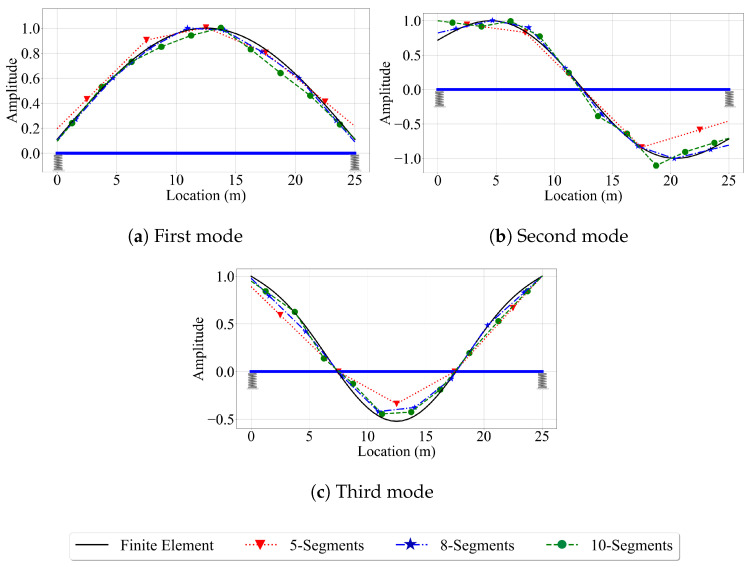
Comparison of the number of segments in constructing mode shapes of the single-span bridge for vehicle speed of 2.5 m/s and sampling frequency of 1000 Hz.

Figure 5 presents the first three mode shape estimates provided by the reference-based SSI method for different number of segments for the velocity of 2.5 m/s and a time step of 0.001 s. Further, also shown in the figure are the exact mode shapes obtained from the finite element analysis and the undeformed shape of the bridge. Here, it should be noted that we positioned the reference car at different locations to achieve the best mode shape estimates for each mode. For the first mode, the reference car was placed at the middle of the bridge while, for the second and third modes, it was located 5 m away from the left support. Once the modal displacements were computed at the middle of each segment, the mode shapes were combined using straight lines. Note that other methods such as cubic spline can also be used for this purpose, but we combined the data points using straight lines because it was used in the original article [31]. The plots in Figure 5 indicate that using eight segments, resulting in 1250 data points per segment for the given vehicle velocity and time step, provided the best estimates for all three mode shapes for the single-span bridge considered in this study. While using five segments failed to provide the spatial resolution to accurately construct the mode shapes, using ten segments led to inaccuracies in the modal coordinates due to the limited number of data points at each segment. Thus, we used eight segments, which leads to a segment length of 3.125 m, in applying the reference-based SSI method for the single-span bridge. For the two-span bridge, the number of segments was increased, but the segment length was kept constant to ensure the number of data points per segment was the same for the two bridges.

**Figure 6:**
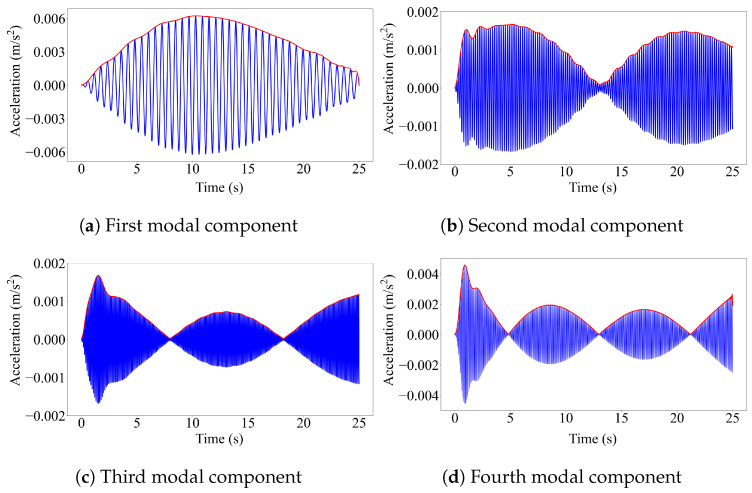
The first four modal components of the single-span bridge obtained from the CP response at 2.5 m/s.

We now look at an example of the application of modal component identification from the contact point response. Figure 6 presents the modal components of the first four modes of the single-span bridge extracted from the contact point response of a quarter-car model using elliptic filter method, as well as the amplitude of the Hilbert transforms (HT) of these components. As shown in the figure, Hilbert transform amplitudes are always positive because of their definition. Thus, in estimating the second and higher mode shapes for a single-span bridge, engineering judgment is required to construct the mode shapes appropriately when half-car [42] or elliptic filter methods [39] are used.

If we compare Figure 5 and Figure 6, we can also observe a major difference between the reference-based SSI method and the other two methods, which are based on modal component estimation. The reference-based SSI method provides modal coordinate estimates at discrete points, and the number of these discrete points is equal to the number of segments used. The mode shapes are then created by combining these discrete modal coordinate estimates, as explained above. On the other hand, the methods that are based on modal component estimation, i.e., the half-car method and the elliptic method, provide continuous mode shape estimates.

#### 4.1.1. Case I: Smooth Road Profile

**Figure 7:**
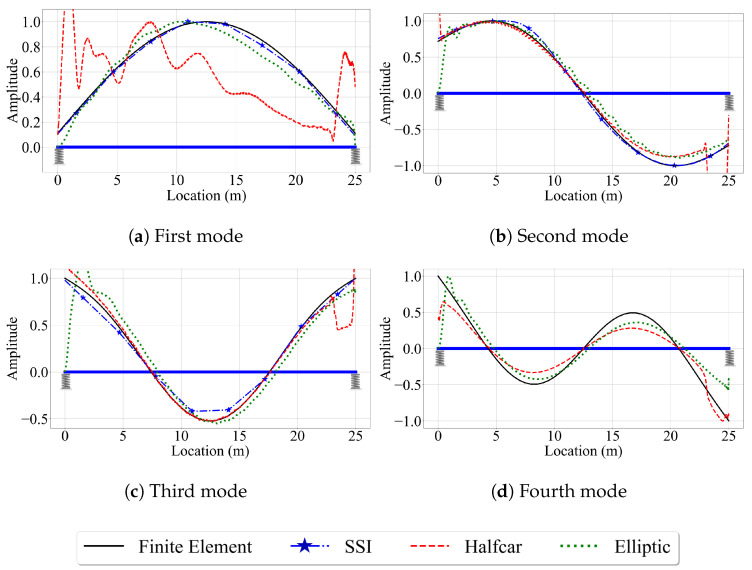
Mode shape estimates for the smooth road profile and v=2.5 m/s.

In this section, we evaluate the efficacy of the three VSMs in estimating the mode shapes of a single-span bridge with a smooth road profile. This case, arguably, presents the simplest case as the vehicle accelerations are not contaminated by the vibrations created by the irregularities on the road. Figure 7 depicts the mode shape estimates for the first four modes of the single-span bridge with smooth road profile and for a vehicle speed of 2.5 m/s. The mode shapes are normalized so that the absolute maximum value is 1.0. The results indicate that all three methods can successfully estimate the second and third mode shapes for the majority of the bridge. Particularly, the reference-based SSI method provides very accurate estimates of the first three mode shapes for the smooth bridge and v=2.5 m/s. However, this method failed to provide sufficient stable modes to recover the fourth mode shape.

**Figure 8:**
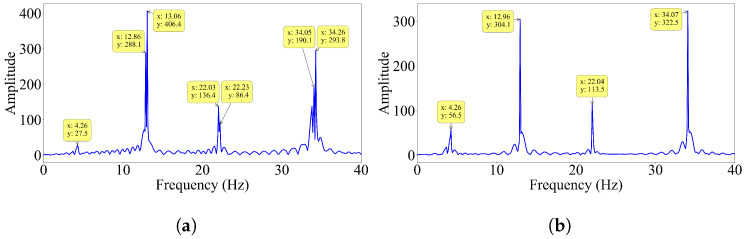
Fourier amplitude spectrum of the (**a**) contact point response for smooth road profile and (**b**) bridge response at mid span V=2.5 m/s.

On the other hand, the half-car method provides very poor mode shape estimates for the first mode, even for the slowest vehicle speed and smooth road profile; see Figure 7a. This is a rather surprising observation because the dynamic behavior of single-span bridges is usually dominated by the first mode response, and the first mode is the easiest mode to identify accurately. To understand the reason for this, we computed the Fourier amplitude spectrum (FAS) of the contact point response, as illustrated in Figure 8a. In addition, also plotted in Figure 8b is the FAS of the mid-span response of the bridge. In both spectra, the first four natural frequencies of the bridge are visible. Strikingly, the energy at the first mode is lower than the energy at the other bridge frequencies in both FAS. Hence, we can state that the bridge has a weak first mode response under the dynamic load provided by the half-car crossing the bridge leading to the inaccurate mode shape estimate for the first mode provided by the half-car method. This can be explained by the interaction between the pitching mode of the half-car model and the first mode of the bridge. These two modes have frequencies that are very close to each other, 3.89 Hz and 4.26 Hz, respectively. Thus, they can be expected to have a significant impact on each other. In the pitching motion of the vehicle, the front and contact points move in the opposite directions. On the other hand, the two points of the bridge, which are connected to the contact points, always move in the same direction when they are vibrating in the first mode. Assuming full contact between the tires of the vehicle and the bridge, this creates a conflict between the pitching motion of the vehicle and the first mode vibration of the bridge weakening the first mode response.

**Figure 9:**
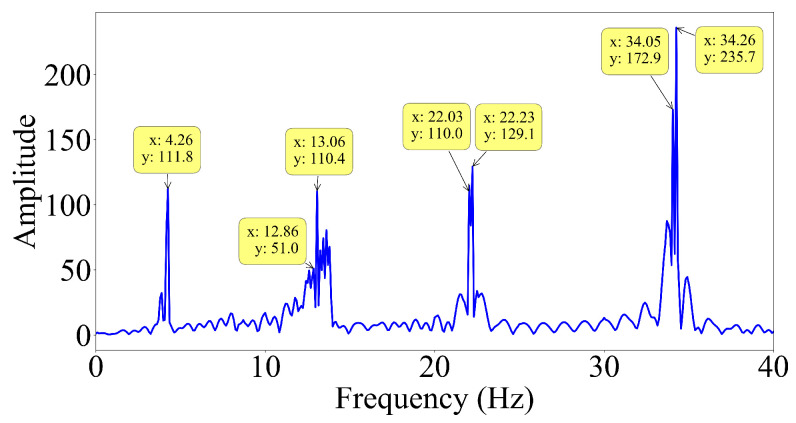
Fourier amplitude spectrum of the contact point response of the modified car for smooth road profile and V=2.5 m/s.

**Figure 10:**
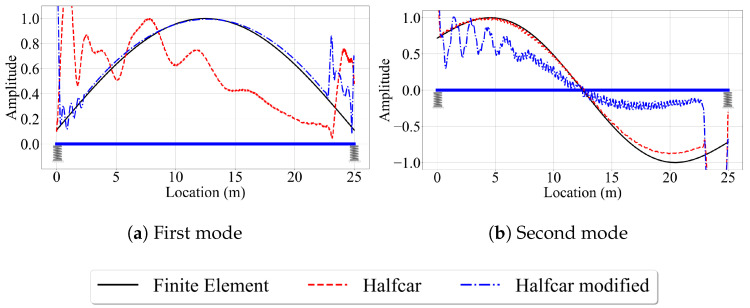
Mode shape estimates by the half-car method for the smooth road profile for two different vehicle frequencies.

To confirm our explanation, we modified the vehicle frequencies such that the pitching frequency does not interfere with the first bridge frequency but interferes with the second frequency. To achieve this, we increased the stiffness of each axle tenfold, while keeping the other properties of the half-car constant. The resulting vehicle frequencies are 8.33 Hz and 12.33 Hz for bouncing and pitching, respectively. Then, we computed the FAS of the contact point response for this case and presented it in Figure 9. In this case, there is significant energy at the first bridge mode frequency but less energy at the second bridge mode frequency. As opposed to the first case, the pitching frequency (12.33 Hz) is close to the second bridge mode frequency (12.96 Hz). Thus, we can expect the pitching motion of the vehicle to interfere with the second mode vibrations of the bridge leading to poorer mode shape estimates. Indeed, when we plot the first and second mode shape estimates provided by the half-car method for the two different vehicle properties, Figure 10, we can observe that, for the modified vehicle properties, the estimated mode shape for the first mode is much improved compared to that obtained for the original vehicle properties. On the other hand, bringing the pitching frequency of the vehicle close to the second mode frequency led to a sharp decline in the accuracy of the second mode shape estimate. Hence, we can conclude that when a half-car model is used, the identification of the bridge mode shapes can be challenging in case the modal frequency is close to the pitching frequency of the vehicle.

**Figure 11:**
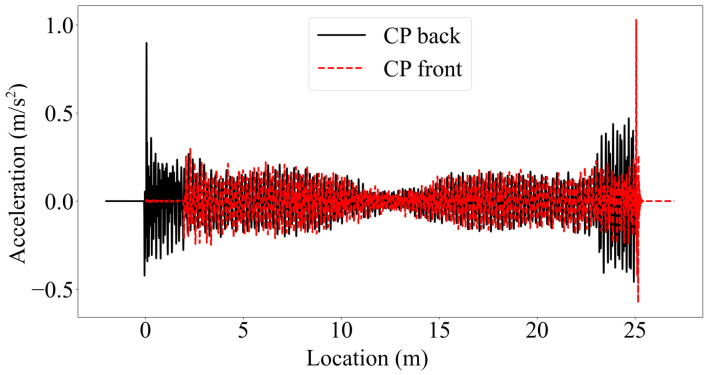
Acceleration time history of the front and rear contact points traveling on a smooth bridge with a velocity of 2.5 m/s.

Further, Figure 7 shows that the mode shape estimates tend to deviate from the finite element solution at the supports, especially for the half-car method, and to some extent, for the elliptic filter method. Thus, we take a closer look at the behavior of the half-car model to try to understand why this model leads to poor mode shape estimates at the edges of the bridge. Figure 11 depicts the front and rear contact point accelerations along the length of the single-span bridge for v=2.5 m/s. When we investigate Figure 11, we can identify two peaks at the two edges of the bridge.

The first one can be observed at the front contact point acceleration when the front axle is 2 m away from the left support. Until the front axle reaches this point, the back axle travels on the approach, which is infinitely rigid compared to the bridge, because the distance between the front and rear axles is 2 m. At the instant the back axle enters the bridge, the bridge deformations change drastically because the total load on the bridge changes, which leads to the acceleration peaks shown in Figure 11. Similarly, at the instant the front axle leaves the bridge (i.e., when the front axle reaches x = 25 m), the total load on the bridge changes once again leading to a sudden change in bridge deformations and a peak at the CP accelerations. Both peaks in the accelerations cause the modal components, and thus the mode shape estimates, to be distorted at the edges. When we reran the same analysis with pin supports instead of elastic supports, we did not observe the same amplification in the CP responses. This is because the pin supports facilitate a much smoother transition from the rigid approaches to the flexible bridge preventing the acceleration peaks. However, when the bridge is seated on elastic supports, the change in the deformations due to an axle entering or exiting a bridge is much more sudden, leading to the acceleration peaks and the distorted mode shapes at the edges.

The elliptic filter method, which also uses modal component identification similar to the half-car method, is not prone to such peak accelerations at the edges because it uses a quarter-car model that does not comprise a transition from a rigid approach to the bridge or vice versa. However, the mode shape estimates of this method are also inaccurate at the edges, especially at the left edge where the vehicle starts its trip. Since the quarter car has only a single axle that acts as the excitation source for the bridge, it is unable to create pre-excitation as the front axle of the half-car does before the back axle enters the bridge. As a result, receiving insufficient dynamic information due to lower vibration levels at the beginning of the quarter-car’s movement leads to a decrease in the accuracy of the extracted modal components, particularly at the left edge of the bridge.

Next, we investigate the effect of vehicle speed on the accuracy of the mode shape estimates of the three VSMs. For this, we focus on the second and third mode shapes, which have relatively high modal displacements at the bearings. In this study, we pay special attention to the mode shape estimates at the ends of the bridge because nearly all the VSM studies that aim to estimate mode shapes of bridges use numerical models with pin supports, but the efficacy of the three methods used in this study in estimating the mode shapes of bridges that are seated on elastic supports remain unexplored. Considering that majority of bridges sit on bearings that have finite stiffness, we focus on the edges of the bridge while evaluating the efficacy of the VSMs.

**Figure 12:**
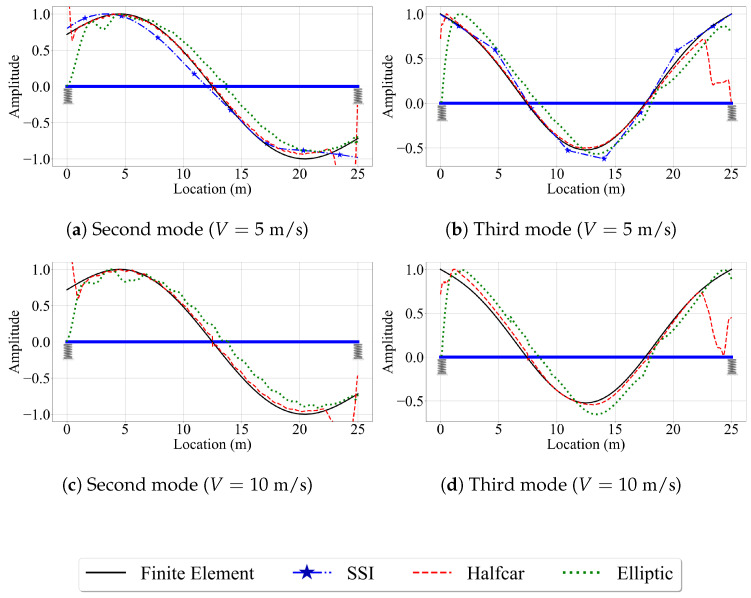
Mode shape estimates for the smooth road profile and V=5 m/s and V=10 m/s.

Figure 12 shows the second and third mode shape estimates from the VSMs, along with the exact mode shapes obtained from the finite element analysis for speeds of 5 m/s and 10 m/s. The first point that attracts our attention is the lack of mode shape estimates for the reference-based SSI method for V=10 m/s. Due to the relatively higher vehicle speed, the number of data points at each of the eight segments used for the reference-based SSI method reduces significantly for V=10 m/s compared to the lower speeds. Therefore, the subspace stochastic identification method fails to identify any stable modes for V=10 m/s and no mode shape estimates are provided by the reference-based SSI method at this speed (Figure 12c,d). Potential remedies to overcome this shortcoming can be using less number of segments at a cost of lowered spatial resolution for mode shapes or using a smaller time step. The latter, however, can be limited by the instrumentation used in real-life applications. On the other hand, for the cases where we can obtain stabilized mode shapes, e.g., for V=5 m/s, the reference-based SSI method provided excellent mode shape estimates along the entire length of the bridge; see Figure 12a,b.

For the half-car method, comparing the estimated mode shapes for different speeds in Figure 7 and Figure 12 indicate that the vehicle speed does not have a significant effect on the accuracy of the mode shape estimates provided by this method when the road profile is smooth. As in the case of V=2.5 m/s, the half-car method provides excellent mode shape estimates between the x=2 m and x=23 m of the bridge for V=5 m/s and V=10 m/s. However, at the edges, the mode shape estimates become inaccurate due to the sudden peaks observed in the front and rear CP accelerations at the start and end of the vehicle’s trip due to the combined effects of elastic support and the half-car model, as explained above.

The effect of speed is more pronounced for the elliptic filter method compared to the half-car method. Although the mode shape estimates are very similar to each other and to the finite element solution for different speeds, especially at the middle of the bridge, the mode shape estimates of the elliptic filter method shift in the travel direction of the bridge as the vehicle speed increases. Since the main difference between the half-car method and the elliptic filter method is the modal decomposition method used to estimate the mode shapes from contact point accelerations, we can state that the main reason for the spatial shift in the mode shape estimates with varying vehicle speeds is likely the properties of the elliptic filter.

In summary, for the smooth road profile, the half-car method stands out among the three VSMs considered in this study as the most robust method against increased vehicle speeds while the reference-based SSI method provides the most accurate mode shape estimates for the cases we can obtain stabilized mode shapes.

#### 4.1.2. Case II: Rough Profile

**Figure 13:**
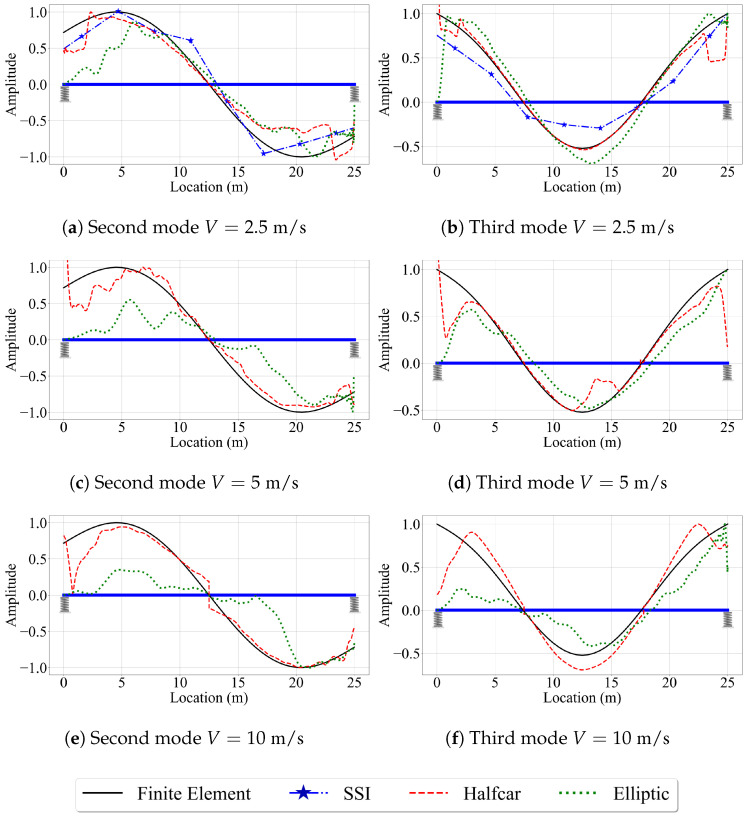
Mode shape estimates for the road profile with roughness for V=2.5 m/s, V=5 m/s, and V=10 m/s.

In order to assess the impact of road roughness on the accuracy of the VSMs, we incorporated this parameter into the numerical analysis. For this, the numerical analysis was repeated for the bridge with the road roughness profile provided in Figure 4. Although we have considered four mode shapes in our study, we focus only on the second and third mode shapes, which have the highest modal displacements at the edges, for brevity. Figure 13 presents the mode shape estimates provided by the VSMs for vehicles that are traveling on a rough road profile with different speeds.

Comparing Figure 13 with Figure 7 and Figure 12 reveal the negative impact of road roughness on the accuracy of the VSMs in estimating the mode shapes. First, due to the road roughness, the reference-based SSI method could not provide stabilized modes for a vehicle speed of 5 m/s leading to an immediate decline in the performance of this VSM compared to the case of smooth road profile, where the reference-based SSI method provided very good mode shape estimates for V=5 m/s; see Figure 12a,b. Further, for the vehicle speed of 2.5 m/s, there is a significant decline in the accuracy of the mode shape estimates provided by each of the three methods. Moreover, the estimates of the elliptic filter method are far from satisfactory for higher vehicle speeds (V=5 m/s and V=10 m/s) when road roughness is considered; see Figure 13c–f. This can be attributed to the fact that the elliptic filter method does not provide any remedy to attenuate the negative effects of road roughness. On the other hand, the half-car method tries to overcome these effects by subtracting the rear CP acceleration from the front CP acceleration [42]. As a result, the half-car method provided the best mode shape estimates, especially for higher vehicle speeds, for the case where road roughness is considered. Noticeably, the shortcomings of the half-car method in estimating the modal coordinates at the edges of the bridge persist when road roughness is considered.

#### 4.1.3. Case III: Rough Profile with Traffic

**Figure 14:**
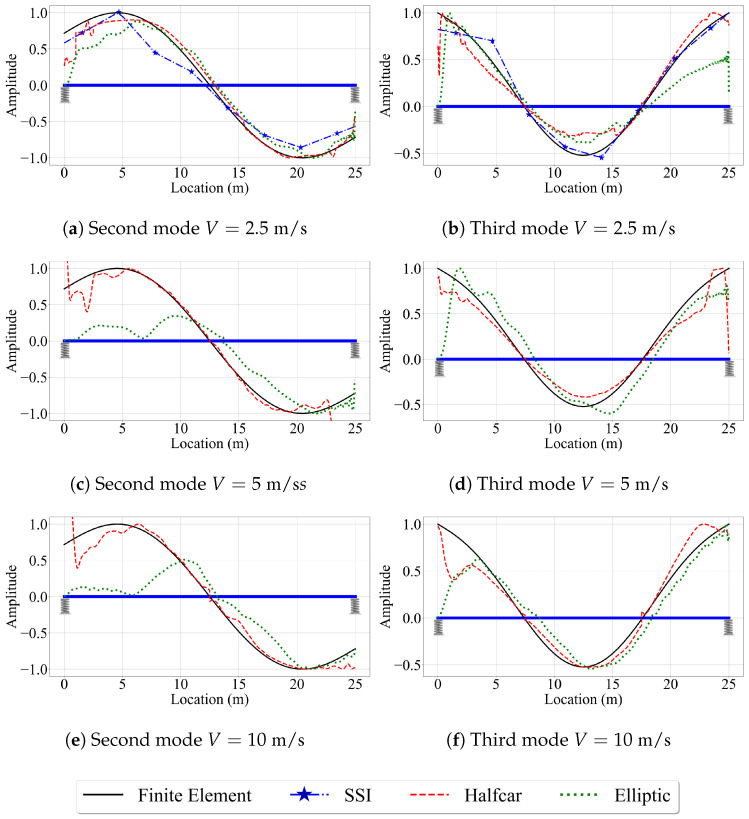
Mode shape estimates for the road profile with roughness and with traffic for V=2.5 m/s, V=5 m/s, and V=10 m/s.

Finally, we investigate the influence of traffic on eliminating the negative effects of road roughness and on the accuracy of the VSMs in mode shape estimation. To pre-excite the bridge, we introduced a leading truck weighing 5t with the same frequency and damping characteristics as the instrumented vehicle. The leading truck travels at the same speed as the instrumented vehicle. The instrumented vehicle enters the bridge once the leading truck travels 40% of the bridge distance. In the simulation, the truck does not continue its motion over the approach slab after reaching the support at the end of the bridge to avoid possible acceleration peaks that may occur in the instrumented vehicle response. The mode shape estimates for the second and third modes provided by the VSMs under these conditions are presented in Figure 14 along with the exact mode shapes.

When comparing Figure 13 and Figure 14, we can observe the positive effects of pre-exciting the bridge on the mode shape estimates obtained by the VSMs. This positive effect arises because the instrumented vehicle enters a bridge which is already in motion, thanks to the dynamic loading induced by the lead vehicle. The increased vibration level of the bridge amplifies the energy content of the bridge modes and enhances the accuracy of the extracted modal components. As such, both the elliptic method and the half-car method benefited from these initial oscillations leading to improved mode shape estimates. On the other hand, the impact of the lead vehicle on the performance of the reference-based SSI method is much more limited. The initial bridge vibrations created by the lead vehicle did not help the reference-based SSI method to obtain stabilized modes for vehicle speeds of 5 m/s and higher. However, the mode shape estimates of the reference-based SSI method for V=2.5 m/s improved with the help of the pre-excitation of the bridge (Figure 14a,b) compared to the case where road roughness is considered without the lead vehicle (Figure 13a,b). The limited positive effect of traffic on the performance of the reference-based SSI method can be explained by the fact that this method uses the vibrations measured on the instrumented vehicle relative to the vibrations measured on the reference vehicle, which is stationary. As such, the positive effects of the vibrations provided by the lead vehicle, which are captured by the CP response, are diminished for the reference-based SSI method because it does not use them directly but in a relative manner.

### 4.2. Two-Span Bridge

The focus of most of the studies on vehicle scanning methods is on single-span bridges. The relatively few studies that consider multi-span bridges are limited to structures that are completely restrained in the vertical directions at the supports [25,26,36,42]. In this study, we provide an objective evaluation of the efficacy of three state-of-the-art vehicle scanning methods in estimating the mode shapes of a two-span bridge that is seated on elastic supports. The details of the bridge are summarized previously in Section 3. The mode shapes of the bridge shown in Figure 3 reveal the main impact of the elastic supports on the bridge behavior. While the modal displacements at the supports remain zero for pin-supported bridges for all mode shapes, the finite stiffness of the elastic supports leads to relatively high modal displacements at these locations. For the two-span bridge, we follow the same outline as the single-span bridge. First, we look at the case where the road roughness is ignored. Next, we investigate the effect of road roughness on the efficacy of the VSMs in estimating mode shapes. Finally, we add a lead vehicle to pre-excite the bridge before the instrumented vehicle enters the bridge. To differentiate these cases from the corresponding cases for the single-span bridge, we name them Cases IV, V, and VI, respectively.

#### 4.2.1. Case IV: Smooth Road Profile

**Figure 15:**
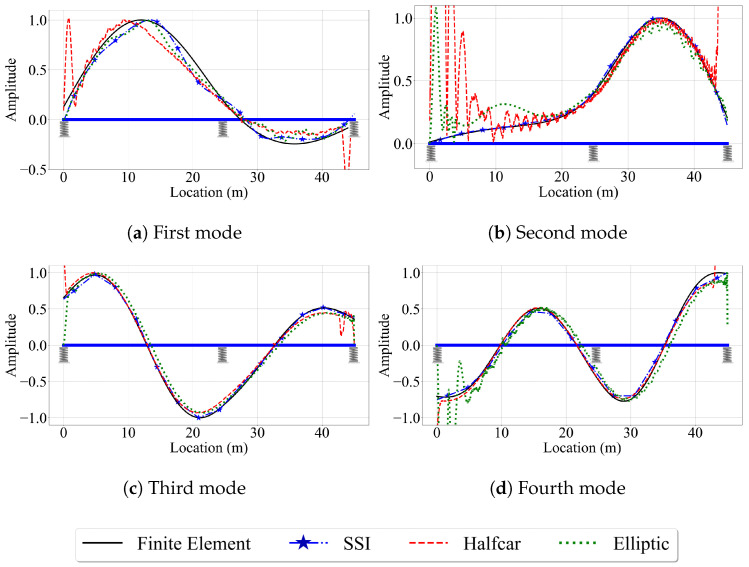
Mode shape estimates for the smooth road profile for V=2.5 m/s for the two-span bridge.

Figure 15 presents the mode shapes estimated by the VSMs when road roughness is not considered and the vehicle is traveling at a speed of 2.5 m/s. The efficacy of the three VSMs in estimating the mode shapes of the two-span bridge, in general, is similar to that of the single-span bridge. The reference-based SSI method provided relatively satisfactory mode shape estimates for the first four bridge modes. Similarly, the half-car and elliptic methods yielded good mode shape estimates, except for the two ends of the bridge. The half-car method suffers from the amplifications in the acceleration amplitude caused by the entrance of the rear axle at the left end of the bridge (the vehicle is traveling towards the right) and the exit of the front axle at the right end of the bridge due to sudden changes in the deformations caused by the elastic support. These amplifications were directly reflected to the modal component estimates provided by the half-car method leading to inaccurate modal displacement estimates in the first and last 2 m of the bridge. On the other hand, although the elliptic method uses a quarter-car model that is not affected by these amplifications, it still struggles to capture the mode shapes at the bridge entrance.

**Figure 16:**
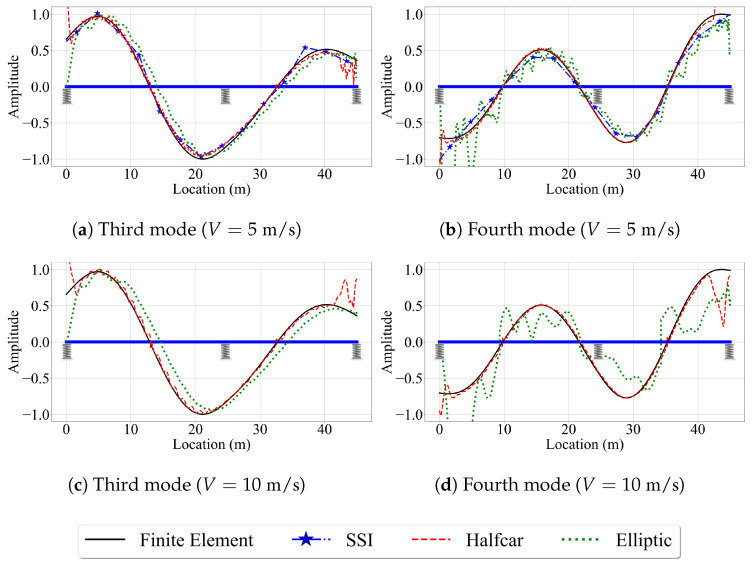
Mode shape estimates for the smooth road profile and V=5 m/s and V=10 m/s for two-span bridge.

Next, we evaluate the effect of vehicle speed on the accuracy of the mode shape estimates. For brevity, we focus only on the third and fourth mode shapes, which have the highest modal displacements at the two ends of the bridge. Figure 16 depicts these mode shapes obtained from the three VSMs for vehicle speeds of 5 m/s and 10 m/s. The effect of vehicle speed for the two-span bridge is similar to its counterpart for the single-span bridge. The most striking effect of higher vehicle speeds is, once again, the lack of mode shape estimates from the reference-based SSI method. Since the segment length was kept constant between the single-, and two-span bridges, the number of data points per segment is constant for a given vehicle speed. As such, the reference-based SSI method failed to obtain stable mode shape estimates for V=10 m/s. However, whenever the reference-based SSI method was successful in obtaining stable modes, it provided mode shape estimates that are relatively accurate; see Figure 15.

Once again, the half-car method was observed to be the most robust method against the effects of vehicle speed. The mode shape estimates provided by the half-car method for the three vehicle speeds considered in this study for the two-span bridge were relatively consistent and accurate except the two ends of the bridge. On the other hand, the elliptic method provided mode shape estimates that become increasingly distorted with an increase in vehicle speed.

#### 4.2.2. Case V: Two-Span Bridge: Rough Profile

**Figure 17:**
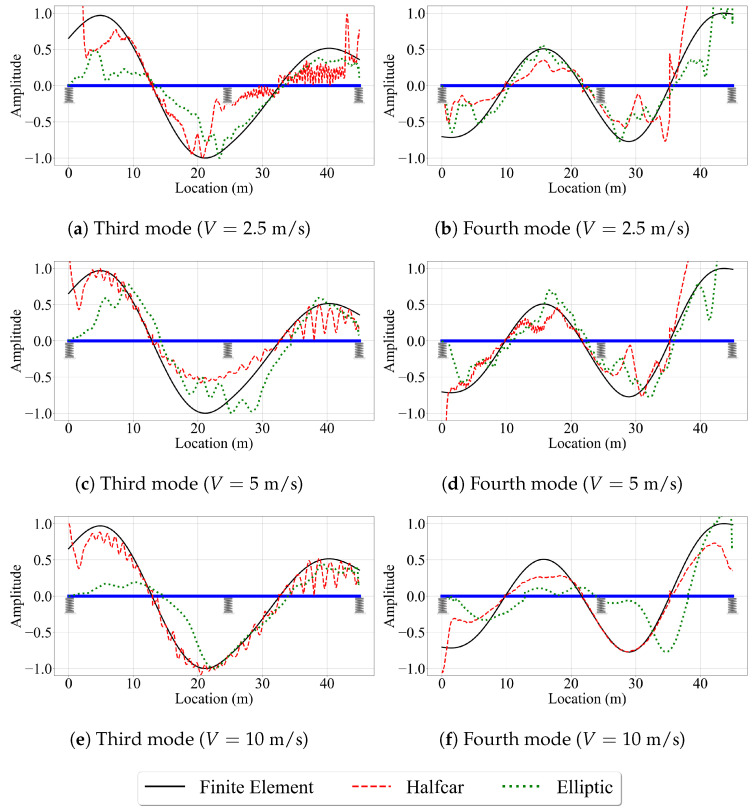
Mode shape estimates for the road profile with roughness for V=2.5 m/s, V=5 m/s, and V=10 m/s for the two-span bridge.

Next, we evaluate the efficacy of the three VSMs in estimating the mode shapes of the two-span bridge under the consideration of road roughness, as simulated by the profile presented in Figure 4. The mode shape estimates provided by the half-car and elliptic methods for the third and fourth mode shapes of the two-span bridge are shown in Figure 17. No mode shape estimates were presented for the reference-based SSI method because this method failed to achieve any stable modes for the two-span bridge, even for the slowest vehicle speed of v=2.5 m/s when road roughness is considered. In general, the mode shape estimates for the two-span bridge provided by the half-car and elliptic methods were also less accurate compared to their counterparts for the single-span bridge when road roughness is considered in the analysis; see Figure 13 and Figure 17. This can be attributed to the additional complexity in the structural behavior provided by the middle support. Although Figure 17 only shows the third and fourth modes for brevity, the results are similar for the first two modes of the two-span bridge. Therefore, it can be stated that the estimation of mode shapes is more challenging for the two-span bridge using VSMs when road roughness is taken into account.

#### 4.2.3. Case VI: Two-Span Bridge: Rough Profile with Traffic

**Figure 18:**
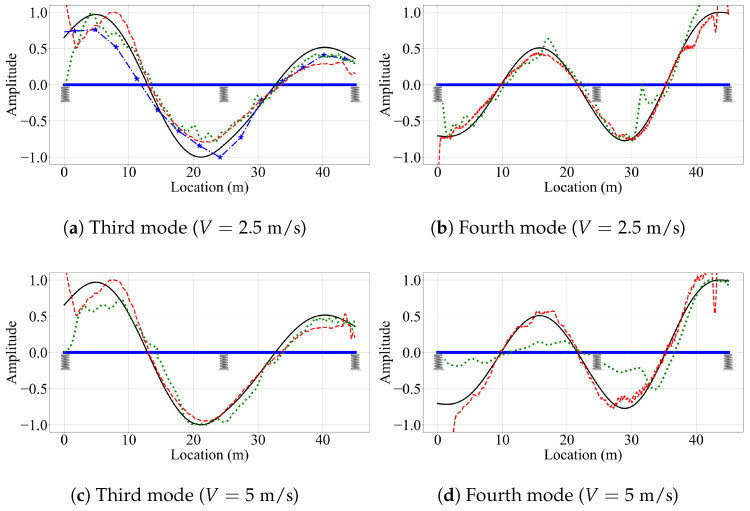

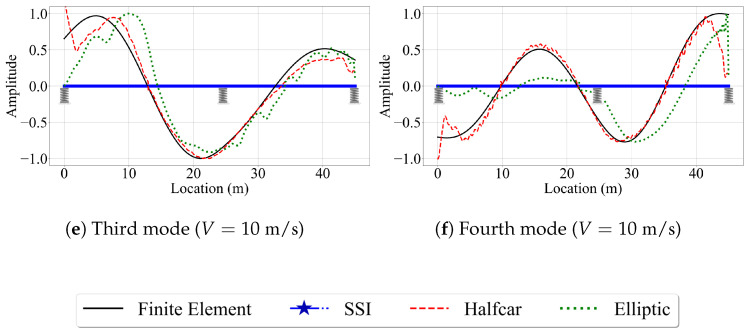
Mode shape estimates for the road profile with roughness and with traffic for V=2.5 m/s, V=5 m/s, and V=10 m/s for the two-span bridge.

In the last case study, we consider the effect of increased oscillations provided by the lead vehicle. The instrumented vehicle starts its trip on the bridge at the moment the lead vehicle reaches a distance of 40% away from the left bearing. Figure 18 depicts the mode shape estimates provided by the three VSMs for the third and fourth mode shapes of the bridge for V=2.5 m/s, V=5 m/s, V=10 m/s. The reference-based SSI method succeeded to provide a mode shape estimate only for the third shape for the slowest vehicle speed considered, V=2.5 m/s. However, for the other cases, the increased bridge oscillations caused by the lead vehicle were not sufficient to provide stable modes for the reference-based SSI method. On the other hand, the increased bridge oscillations created by the lead vehicle significantly improved the mode shape estimates obtained by the half-car method. Comparing Figure 15, Figure 17 and Figure 18, we can state that the increased bridge oscillations induced by the lead vehicle helped mitigate the negative effects of the road roughness, resulting in mode shape estimates that closely resemble those obtained for a smooth road profile. The mode shape estimates provided by the elliptic method are also improved to some extent, although not to the same extent as the half-car method. This difference can be attributed to the fact that the half-car method actively attempts to minimize the adverse effects of road roughness by subtracting the rear contact point response from the front contact point response, whereas the elliptic method lacks such a remedy. However, the oscillations created by the lead vehicle are not enough to overcome the amplifications in the acceleration response observed at the two ends of the bridge and their direct impact on the mode shape estimates at the ends of the vehicle observed for the half-car method and, as a result, the half-car method failed to provide satisfactory estimates for the modal displacements at the two ends of the bridge.

## 5. Concluding Remarks

In this study, we explored the efficacy of three vehicle scanning methods in estimating the mode shapes of bridges that are seated on elastic supports. Although elastic supports are ubiquitous in bridges, the majority of the studies on VSMs use bridge models with pin supports. Via numerical analysis conducted on one single- and one two-span bridge, considering smooth and rough road profiles, and presence of a lead vehicle, we evaluated the accuracy of the mode shape estimates with a special focus on the edges of the bridge. As a result of these analyses, we can draw the following conclusions.

- The reference-based SSI method provides relatively accurate mode shape estimates whenever it succeeds in obtaining stable modes. However, for the majority of the cases we considered, it has failed to provide any mode shape estimates. Specifically, this method is susceptible to the negative effects of higher vehicle speeds and road roughness. Due to its sensitivity to the number of data points available at each bridge segment, segmentation of the bridge needs to be carefully conducted, considering the sampling rate and the vehicle speed.
- The half-car method is robust against the negative effects of vehicle speed and yields improved mode shape estimates, even at high speeds. It successfully alleviates the negative effects of road roughness. However, it may result in inaccurate mode shape estimates when the pitching frequency of the vehicle, which is not present in a quarter-car model, interferes with any bridge mode.
- The elastic supports and using a half-car model provide a combined effect that leads to a significant decrease in the mode shape estimates provided by the half-car method at the edges of the bridge. Due to a change in the total load carried by the bridge at the instant of an axle entering or leaving a bridge via an elastic support, the bridge displacement profile changes suddenly leading to amplifications in the CP accelerations. These amplifications then distort the modal components identified using variable decomposition method leading to inaccurate mode shape estimates at the bridge ends. Considering that the majority of the bridges are seated on elastic supports and the likelihood of using a vehicle with two or more axles in vehicle scanning applications, it is imperative to include this combined effect in future VSM studies.
- Elliptic method yields mode shape estimates that are relatively similar to the half-car method. However, it exhibits a shift in the identified mode shapes as the vehicle speed increases and is negatively affected by road roughness. Using a quarter-car provides this method with an advantage in numerical analysis as it avoids potential negative effects caused by the pitching frequency, which are experienced when a half-car model is used.
- The number of spans, despite the presence of an elastic support at the middle support, did not affect the efficacy of the VSMs because the results for the single-span bridge and the two-span bridge are comparable to each other with no significant impact from the middle support.
- Based on the presented results and the two previous items discussed in the conclusions, we can state that utilizing a quarter-car model can offer advantages. However, these observations only reinforce the need to accurately model the vehicle in the numerical models used to assess or develop VSMs. If a single car with two axles is used in the field applications, using a quarter-car model in numerical analysis can potentially hide the problems that can be encountered when the method is used in the field. Thus, it is imperative for any numerical study to accurately model the instrumented vehicle and the vehicle.

The numerical study summarized herein highlights the need for future research to increase the efficacy of the VSMs in estimating the mode shapes of bridges. The results especially shed light on the importance of accuracy of the numerical models used. While studies that use quarter-car models and pin-supported bridges contribute to our understanding of vehicle scanning methods and pave the way for improved methods, they are likely to encounter challenges in field applications as they cannot emulate the conditions in the field. Further, the need for experimental studies is also highlighted because only through experimental studies that emulate real-life conditions or through field applications can we create a complete picture of the challenges related to vehicle scanning applications.

## Figures and Tables

**Table 1:** The properties of bridge and vehicles.

**Bridge**	Mass per length	ρ=2 t/m
	Young’s modulus	E=30 GPa
	Moment of inertia	I=0.20 m${}^{4}$
	Spring coefficient	$k_{bridge}=10^{5}$ kN/m
**Quarter-car**	Mass	$m_{v}=2$ t
	Stiffness coefficient	$k_{v}=550$ kN/m
	Damping coefficient	$c_{v}=2$ kN·s/m
	Bounce frequency	$f_{v,q}=2.63$ Hz
**Half-car**	Mass	$m_{v}=2$ t
	Mass moment of Inertia	$J_{v}=1.4$ t·m${}^{2}$
	Stiffness coefficient (front)	$k_{1}=480$ kN/m
	Stiffness coefficient (rear)	$k_{2}=240$ kN/m
	Damping coefficient (front)	$c_{1}=1$ kN·s/m
	Damping coefficient (rear)	$c_{2}=1$ kN·s/m
	Axle distance to the mass	$d_{1}=d_{2}=1$ m
	Bounce frequency	$f_{v,h}=2.63$ Hz
	Pitching frequency	$f_{v,r}=3.89$ Hz
**Truck** (in traffic)	Mass	$m_{t}=5$ t
	Stiffness coefficient	$k_{t}=1370$ kN/m
	Damping coefficient	$c_{t}=2$ kN·s/m
	Bounce frequency	$f_{v,t}=2.63$ Hz

## Data Availability

No new data were created or analyzed in this study. Data sharing is not applicable to this article.
